# Cardiomyocyte‐specific overexpression of FPN1 diminishes cardiac hypertrophy induced by chronic intermittent hypoxia

**DOI:** 10.1111/jcmm.18543

**Published:** 2024-07-25

**Authors:** Ji‐Xian Song, Shan Xu, Qi Chen, Yujing Gou, Chen‐Bing Zhao, Cui‐Ling Jia, Han Liu, Zhi Zhang, Bo‐liang Li, Yuhui Gao, Yashuo Zhao, En‐Sheng Ji

**Affiliations:** ^1^ Hebei Technology Innovation Center of TCM Combined Hydrogen Medicine Hebei University of Chinese Medicine Shijiazhuang China; ^2^ Department of Physiology, Institute of Basic Medicine Hebei University of Chinese Medicine Shijiazhuang China; ^3^ Hebei Key Laboratory of Turbidity Toxin Syndrome The First Affiliated Hospital Hebei University of Chinese Medicine Shijiazhuang China

**Keywords:** cardiac dysfunction, ferroportin 1, iron, oxidative damage

## Abstract

The significance of iron in myocardial mitochondria function cannot be underestimated, because deviations in iron levels within cardiomyocytes may have profound detrimental effects on cardiac function. In this study, we investigated the effects of ferroportin 1 (FPN1) on cardiac iron levels and pathological alterations in mice subjected to chronic intermittent hypoxia (CIH). The cTNT‐FPN1 plasmid was administered via tail vein injection to induce the mouse with FPN1 overexpression in the cardiomyocytes. CIH was established by exposing the mice to cycles of 21%–5% FiO_2_ for 3 min, 8 h per day. Subsequently, the introduction of hepcidin resulted in a reduction in FPN1 expression, and H9C2 cells were used to establish an IH model to further elucidate the role of FPN1. First, FPN1 overexpression ameliorated CIH‐induced cardiac dysfunction, myocardial hypertrophy, mitochondrial damage and apoptosis. Second, FPN1 overexpression attenuated ROS levels during CIH. In addition, FPN1 overexpression mitigated CIH‐induced cardiac iron accumulation. Moreover, the administration of hepcidin resulted in a reduction in FPN1 levels, further accelerating the CIH‐induced levels of ROS, LIP and apoptosis in H9C2 cells. These findings indicate that the overexpression of FPN1 in cardiomyocytes inhibits CIH‐induced cardiac iron accumulation, subsequently reducing ROS levels and mitigating mitochondrial damage. Conversely, the administration of hepcidin suppressed FPN1 expression and worsened cardiomyocyte iron toxicity injury.

## 1 INTRODUCTION

Obstructive sleep apnoea (OSA) is characterized by the collapse of the upper respiratory tract during sleep, resulting in recurrent interruptions in respiration and induction of chronic intermittent hypoxia (CIH).^1^ The American Heart Association has indicated that CIH is as an independent risk factor for various cardiovascular diseases and can increase the incidence and fatality rates of arrhythmia, hypertension and coronary heart disease.^2^ Continuous positive airway pressure ventilation (CPAP) therapy has been demonstrated to enhance left ventricular ejection fraction (EF) and decrease systolic blood pressure. However, the patients exhibited poor treatment adherence. Therefore, more efficacious interventions are needed to enhance the survival of patients with OSA and mitigate the risk of cardiovascular diseases.

Previous studies demonstrated a correlation between cardiac hypertrophy and OSA.^3^ Pathological cardiac hypertrophy, characterized by an increase in the size of cardiomyocytes and an overall increased rate of protein synthesis, plays a significant role in the development of systolic dysfunction, arrhythmias and sudden death.^4^ A meta‐analysis of echocardiographic studies has demonstrated an association between the severity of OSA and an increased likelihood of left ventricular hypertrophy (LVH).^5^ Furthermore, animal studies have revealed that CIH exposure can induce myocardial damage accompanied by systolic and diastolic dysfunctions, and that the underlying mechanisms may be associated with oxidative stress, inflammatory responses and iron overload.^6^, ^7^ In addition, excessive iron exacerbated age‐related cardiac hypertrophy in a mouse model of haemochromatosis (HH).^8^

Our previous studies have shown that CIH can cause iron deposition in various organs of mice, such as the heart, brain and kidneys.^6^, ^9^, ^10^ Maintaining iron homeostasis is crucial for the optimal performance of all types of mammalian cells, with particular significance for preserving cardiovascular health.^11^ Excessive iron levels may contribute to the development of LVH and ferroptosis, ultimately leading to cardiac dysfunction in mice lacking the ferritin light chain (FTH).^12^ Iron deposition has been observed to lead to decreased myocardial contractility in patients with severe beta‐thalassaemia; similar cardiac dysfunction has been observed in mouse models of iron overload.^13^, ^14^

Iron metabolism in cardiomyocytes is primarily facilitated by its uptake, storage and release.^15^ Myocardial iron intake is mainly dependent on divalent metal transporter 1 (DMT1) and transferrin receptor 1 (TfR1). Ferroportin 1 (FPN1), the only cellular iron efflux protein, is composed of two 6‐times transmembrane helical bundles (N and C lobes) and is prominently expressed in cardiomyocytes.^16^, ^17^, ^18^ Hepcidin, a liver‐derived antibacterial polypeptide and central iron‐regulatory factor, regulates the expression of FPN1 at the post‐transcriptional level, which in turn regulates systemic or intracellular iron levels.^19^, ^20^, ^21^ Studies have shown that cardiac‐specific FPN1 knockout mice exhibit an imbalance in myocardial iron metabolism and cardiac insufficiency, whereas cardiomyocyte‐specific knockout of hepcidin leads to the deregulation of cardiac FPN1 and lethal iron deficiency due to increased iron efflux, suggesting an important role for the hepcidin‐FPN1 axis in the regulation of iron cardiomyocyte homeostasis.^22^, ^23^, ^24^ However, our understanding of the alterations and regulatory pathways of FPN1 during CIH is limited. Although some studies have suggested a potential association between the mechanism of hypoxia/reoxygenation injury in cardiomyocytes and iron homeostasis,^25^ the specific role of FPN1 in this process remains unclear.

Therefore, to ascertain the role of cardiac FPN1, we established a mouse model with FPN1 overexpression in vivo and administered exogenous hepcidin to H9C2 cells to simulate the FPN1‐deficiency model in vitro. Furthermore, we investigated whether FPN1 expression affected CIH‐induced iron accumulation and myocardial injury.

## 2 MATERIALS AND METHODS

### 2.1 Reagents and antibodies

The reagents used were an adeno‐associated virus (AAV) plasmid (Hanbio, Shanghai, China), hepcidin‐25 peptide (ab31875; Abcam, Cambridge, MA, USA), deferoxamine mesylate (DFO, D873692; MACKLIN, Shanghai, China) and ferric ammonium citrate (FAC, F5879, Sigma‐Aldrich, St. Louis, MO, USA). The following kits were used: TUNEL kit (A112, Vazyme Biotech, Nanjing, China), dihydroethidium kit (DHE, 19709, Cayman Chemical, Ann Arbor, Michigan, USA), JC‐1 mitochondrial membrane potential (MMP) detection kit (C2005, Beyotime Biotech Inc, Shanghai, China), total superoxide dismutase kit, malondialdehyde, catalase (T‐SOD, MDA, and CAT; A001‐1, A003‐1, and A007‐1; Nanjing Jiancheng Bioengineering Institute, Jiangsu, China), serum and tissue iron assay kits (A039‐1‐1 and A039‐2‐1, Nanjing Jiancheng Bioengineering Institute, Jiangsu, China), RNA extraction kit (DP419, Tiangen Biotech, Beijing, China), PrimeScript TM RT Reagent Kit with gDNA Eraser and SYBR‐Green PCR Master Mix kit (RR047A and RR820A, Takara, Dalian, China), FerroOrange probe (Maokangbio, MX4580, Shanghai, China), Mitochondrial Membrane Potential and Apoptosis Detection Kit (C1071M, Beyotime Biotech Inc, Shanghai, China), Annexin V‐FITC/PI Double Staining Apoptosis Detection Kit (SC123‐02, SEVEN Biotech, Beijing, China), Minute™ Mitochondrial Isolation Kit (MP‐007, invent, Beijing, China), Mitochondrial Complex I and II Kit (FHTA‐1‐Y and FHTB‐2‐Y, Cominbio, Suzhou, China) and BODIPY™ 581/591 C11 (D3861, Thermo Scientific, former Savant, MA, USA).

The following antibodies were used: FPN1 (MTP11‐A, Alpha Diagnostic International, International, San Antonio, TX, USA), mitochondrial fission protein 1 (Fis‐1), dynamin‐related protein 1 (Drp‐1), and optic atrophy 1 ferritin light chain (Opa‐1) (505821, 382977, and 382025; Zen‐bioscience, Chengdu, China), Bcl‐2 (YT0470, Immunoway, NH, USA), caspase 3 and iron regulatory proteins 1/2 (IRP‐1/2) (#14220 and #20272S/#37135S, Cell Signaling Technology, Danvers, MA, USA), nicotinamide adenine dinucleotide phosphate oxidase 2 (NOX2) and α‐Tubulin (GTX56278 and GTX628802, GeneTex, San Antonio, TX, USA), 4‐hydroxynonenal (4‐HNE, ARG70025, Arigo Biolaboratories, Hsinchu, Taiwan), FTH and ferritin light chain (FTL) (ab183781 and ab218400, Abcam, Cambridge, MA, USA), hepcidin (DF6492, Affinity, Cincinnati, OH, USA), cardiac troponin T (cTNT), Bax, and GAPDH (GB113806, GB11007, and GB15002, Servicebio, Wuhan, China).

### 2.2 Animals

BALB/c mice (SPF grade, male, 20–22 g) were purchased from Beijing Vital River Laboratory Animal Technology Co., Ltd., Beijing, China. Animals were housed in the animal centre with controlled temperature, light and ventilation and fed with standard chow and water. All mice were allowed to acclimate to their environment for 1 week before modelling. Animal handling was performed according to the National Guidelines for the Management and Use of Laboratory Animals and was approved by the Ethical Review Committee for Animal Experiments of Hebei University of Traditional Chinese Medicine (Animal Ethics Number: DWLL2021097).

BALB/c mice (*n* = 40) were randomly divided into WT, cTNT‐FPN1, CIH and CIH + cTNT‐FPN1 groups. A CIH mouse model was established to simulate OSA. The mice were placed in a controlled hypoxic chamber (OxyCycler, BioSpherix Ltd., Parish, NY, USA), in which the O_2_ concentration was reduced from 21% to 5% over an initial period of 1.5 min and then returned to 21% over a period of 1.5 min. The cycle was repeated every 3 min for 8 h each day (9:00–17:00). Mice in the WT and cTNT‐FPN1 groups were placed in the same chamber with normal air.^6^ The CIH and CIH + cTNT‐FPN1 groups were subjected to CIH for 28 days.

The cTNT‐FPN1 plasmid was injected into the tail vein of FPN1‐overexpressing mice. The FPN1 adeno‐associated viral plasmid AAV9‐cTNT‐FPN1 (≥1 *×* 10^12^ v.g/mL, Hanbio Biotechnology, Shanghai, China) was constructed, which contains upstream sequences of cardiomyocyte promoter (cTNT) + *Slc40a1* gene (*FPN1*) labelled with ZsGreen. cTNT‐FPN1 plasmid (100 μL) was injected into mice via the tail vein, and control mice (WT) were injected with a blank plasmid.^26^ After 4 weeks, the heart tissue was collected and prepared into frozen sections, and the intensity of FITC fluorescence was observed under a microscope to verify the success of the model. Subsequently, the AAV9‐cTNT‐FPN1 plasmid was injected every 2 weeks to enable sustained high expression in cardiomyocytes.

### 2.3 Echocardiography

Mice cardiac ultrasound was performed using an MS‐250 probe (Vevo 2100, Visualsonics Inc., Toronto, Canada) on Day 27 of CIH exposure. First, mice were anaesthetised with 2% isoflurane, after which they were placed on an operating table and epilated.^27^ Subsequently, a coupling agent was applied to the cardiac region, and the ultrasound probe was tightly fitted to the skin to detect the relevant indices. The EF, fractional shortening (FS), left ventricular end‐diastolic volume (LVEDV) and left ventricular posterior wall depth (LVPW) were measured using M‐mode recordings of the short‐axis view.

### 2.4 Cardiac enzyme test

The serum levels of lactic dehydrogenase isoenzyme (LDH) and lactic dehydrogenase isoenzyme 1 (LDH1) were determined in each group of mice using an Automatic Biochemical Analyzer (iChem‐530*, ICBIO, Shenzhen, China).

### 2.5 HE staining

Haematoxylin and eosin (HE) staining was performed to observe the basic structure of cardiac tissue. Cardiac paraffin sections (5 μm) were dewaxed with xylene and rehydrated with a gradient of alcohol. The sections were stained with haematoxylin, differentiated with hydrochloric acid‐ethanol, stained with eosin, dehydrated with a gradient of alcohol and vitrified with xylene.

### 2.6 Masson staining

Masson staining was used to detect fibrosis in the cardiac tissue. After dewaxing and rehydration, sections were sequentially stained with haematoxylin, hydrochloric acid, ethanol, Masson blue liquid, distilled water, ponceau, phosphomolybdic acid and aniline blue. The sections were dehydrated with 95% alcohol and anhydrous ethanol, hyalinized with xylene and sealed with neutral gum. The area ratio of fibrotic cells was analysed using Image‐Pro Plus Software (version 6.0; Media Cybernetics, USA).

### 2.7 WGA staining

WGA staining specifically labels the cardiomyocyte membrane and is used to assess structural abnormalities in cardiomyocytes. Paraffin sections were deparaffinized and rehydrated, followed by treatment with EDTA antigen repair solution. Subsequently, the sections were washed and incubated with the WGA solution (a cell surface stain, 1:200) for 60 min at 37°C in the dark. Sections were incubated with DAPI for 5 min at room temperature (RT). The sections were sealed using an anti‐fade sealer, examined and imaged using a fluorescence microscope.

### 2.8 Transmission electron microscopy

Transmission electron microscopy (TEM) was used to observe the mitochondrial ultrastructure in cardiac tissues. After deep anaesthesia, we used a blade to quickly cut and harvest fresh cardiac tissue blocks of no more than 1 mm^3^ and placed the tissues in an electron microscope fixative. After parcelling with resin, the blocks were cut into 60‐ to 80‐nm thick sections using an ultramicrotome and stained with uranyl acetate and lead nitrate. Images were captured using a HITACHI HT7800 electron microscope (Hitachi High‐Technologies Corp., Tokyo, Japan).

### 2.9 Mitochondrial membrane potential measurement

First, the mitochondria were extracted from the cardiac tissues. Then, the JC‐1 MMP detection kit was used to detect MMP, following the manufacturer's instructions. Fluorescence was measured using a multifunctional microplate reader at *E*_*m*_ = 425–520 nm, *E*_*x*_ = 485 nm (Varioskan LUX, Thermo Fisher Scientific, former Savant, MA, USA).

### 2.10 TUNEL staining

TUNEL staining was used to assess apoptosis in cardiac tissue. After dewaxing and rehydration, the sections were rinsed with phosphate buffer (PBS, 0.01 M, pH 7.4). Then, a protease K solution (20 μg/mL) was incubated for 20 min at RT. After washing with PBS and equilibration, the sections were incubated with a reaction mixture of bright green labelling mixture (containing recombinant TdT enzyme) at 37°C for 60 min. DAPI staining was performed for 5 min at RT and the sections were sealed with an anti‐fluorescent bursting agent. The fluorescence of FITC and DAPI was detected at 488 and 460 nm, respectively. The total number of apoptotic cells was counted using Photoshop CS version 5.0 software (AdobeSystems Inc., California, USA).

### 2.11 DHE staining

DHE staining was used to detect the level of reactive oxygen species (ROS) in cardiac tissues. Cryosections of the cardiac tissue were brought to RT and washed with PBS. Sections were incubated with a fluorescence‐quenching agent to remove background fluorescence. Then, 5 μM DHE was added to the slices and incubated at 37°C for 30 min. After washing, sections were re‐stained with DAPI for 5 min at RT. Finally, the sections were sealed with an anti‐fluorescence quencher and visualized under a fluorescence microscope.

### 2.12 Oxidative stress‐related products

An appropriate amount of heart tissue was homogenized and centrifuged and the supernatant was collected to obtain a homogenate (10% g/V). The protein concentration was measured using the BCA method. The activity of T‐SOD and CAT, and the MDA content were determined using commercial kits according to the manufacturer's instructions. Finally, the T‐SOD, CAT and MDA levels were measured at 550, 405 and 532 nm, respectively, using a multifunctional microplate reader.

### 2.13 Perls' staining

Perls' staining was used to detect the trivalent iron content and distribution in the cardiac tissue. After deparaffinization and rehydration, the sections were incubated with 3% H_2_O_2_ for 20 min at RT, and then soaked in a newly configured Prussian blue reaction solution (1%) containing potassium ferricyanide and hydrochloric acid for 6 h at RT. After washing with PBS, the sections were enhanced using a DAB kit, re‐stained with haematoxylin, dehydrated with gradient alcohol, made transparent with xylene and sealed with a neutral resin. The resulting images were visualized under a microscope, and the mean density of the iron content was calculated using ImageJ software (National Institutes of Health, Bethesda, MD, USA).

### 2.14 Determination of total iron

The total iron content was determined according to the manufacturer's specifications. Approximately 50 mg of cardiac tissue was weighed and mixed with nine times the volume of saline. Mechanical homogenization was performed in an ice‐water bath followed by centrifugation. The collected supernatant was combined with three times the volume of iron colourant and thoroughly mixed. The mixture was then incubated at a temperature of 95°C for 5 min. After cooling and centrifuging, the absorbance of the supernatant was measured at 520 nm. Total iron content was calculated using the relevant formula.

### 2.15 Immunological staining

Paraffin sections were dewaxed, rehydrated and incubated with 3% H_2_O_2_ to remove endogenous peroxidases. The sections were subjected to antigen retrieval at high temperature using citrate antigen repair buffer (10 mM, pH 6.0). Sections were blocked with 10% goat serum for 1 h at 37°C.

For immunohistochemistry (IHC) staining, the cardiac sections were incubated with hepcidin primary antibody overnight at 4°C. On the second day, sections were incubated with HRP‐conjugated second antibody for 1 h at 37°C. The sections were then enhanced and stained using a DAB kit. After sealing with a neutral resin, images were acquired, and the mean density of positive cells was calculated using IPP 6.0 software (version 6.0; Media Cybernetics, USA).

For immunofluorescence double staining, the cardiac sections were incubated with primary antibodies FPN1 and cTNT or hepcidin overnight at 4°C after clear autofluorescence. On the second day, fluorescent secondary antibodies against the appropriate species were added and the sections were sealed with an anti‐fluorescence quenching sealer containing DAPI. Finally, sections were visualized under a fluorescence microscope.

### 2.16 Q‐PCR

Total RNA was extracted from the heart tissue using an RNA extraction kit. The extracted total RNA (1 μg) was reverse transcribed into cDNA using the PrimeScript™ RT Reagent Kit with gDNA Eraser. PCR amplification was performed using SYBR Green PCR Master Mix kit. The expression levels of *FPN1*, atrial natriuretic peptide type A (*Nppa*), natriuretic peptide type B (*Nppb*), myosin heavy chain 7 (*MYH7*), *Fis‐1*, *Opa‐1*, *Drp‐1*, *FTL*, *FTH* and *hepcidin* mRNA were determined by quantitative PCR (Q‐PCR; Bio‐Rad, CA, USA). The primer sequences used for Q‐PCR are listed in Table 1. The relative gene expression was calculated using the 2^−ΔΔCt^ formula.

**TABLE 1:** The sequence of primers used for the expression of genes.

Gene	Forward	Reverse	Length
*β‐Actin*	AGGCCCAGAGCAAGAGAGGTA	TCTCCATGTCGTCCCAGTTG	81 bp
*Nppa*	GGGTAGGATTGACAGGATTGG	CCTCCTTGGCTGTTATCTTC	79 bp
*Nppb*	ATCCGTCAGTCGTTTGGG	CAGAGTCAGAAACTGGAGTC	84 bp
*MYH7*	TGTTTCCTTACTTGCTACCC	GGATTCTCAAACGTGTCTAGTG	115 bp
*Fis‐1*	AATATGCCTGGTGCCTGGTT	GCTGTTCCTCTTTGCTCCCT	102 bp
*Drp‐1*	AGGTTGCCCGTGACAAATGA	TCAGCAAAGTCGGGGTGTTT	86 bp
*Opa‐1*	GTGACTATAAGTGGATTGTGCCTG	AACTGGCAAGGTCTTCTGAGC	105 bp
*FPN1*	TGGATGGGTCCTTACTGTCTGCTA	TGCTAATCTGCTCCTGTTTTCTCC	139 bp
*FTL*	ACAGCCCAGGAGACCTTAAGAACA	ACCTTTGAACAAGCTCACCTCCGA	97 bp
*FTH*	CGCCCAGATTTTACACAGTG	TTGGAGTGTCGGTGCTTAAA	91 bp
*Hepcidin*	AGACATTGCGATACCAATGCA	GCAACAGATACCACACTGGGAA	108 bp

### 2.17 Western blot

Initially, pre‐cooled RIPA lysate was added to the heart tissue or H9C2 cells, followed by homogenization and centrifugation. Subsequently, protein concentration was assessed using the BCA method. Proteins were separated using sodium dodecyl sulphate–polyacrylamide gel electrophoresis and transferred to polyvinylidene fluoride (PVDF) membranes. Following the application of a 5% skim milk powder block, the blots were incubated with primary antibodies overnight at 4°C, including FPN1, Fis‐1, Drp‐1, Opa‐1, Bcl‐2, Bax, caspase 3, NOX‐2, 4‐HNE, FTH, FTL, IRP‐1, IRP‐2, α‐Tubulin and GAPDH. The following day, blots were incubated with horseradish peroxidase‐conjugated secondary antibodies for 2 h at RT. Finally, the immunoreactive proteins were imaged using the ECL method. ImageJ software (National Institutes of Health, Bethesda, MD, USA) was used to analyse the mean grey value of the immunoreactive bands.

### 2.18 Cell viability assay

H9C2 cells were cultured in DMEM supplemented with 10% fetal bovine serum (FBS), penicillin and streptomycin at 37°C with 5% CO_2_. A cell model of intermittent hypoxia (IH) was established for 24 h, consisting of each cycle of 0.1% O_2_ for 3 min, followed by 21% O_2_ for 7 min. In the control group, cells were cultivated under normal oxygen conditions.^28^ The cells were treated with hepcidin, FAC and DFO.^29^ At the end of the treatment, cell viability, protein blot analysis and other assays were performed.

Cell viability was evaluated using Cell Counting Kit‐8 (CCK‐8). H9C2 cells were seeded at a density of 1 × 10^4^ cells per well in 96‐well culture plates. At the end of the processing, CCK‐8 reagent was added to the plates and incubated at 37°C for 1 h. Subsequently, the absorbance was detected at 450 nm using a multifunctional microplate reader (Varioskan LUX, Thermo Fisher Scientific).

### 2.19 LIP levels of H9C2 cells

A FerroOrange probe was used to assess the labile iron pool (LIP, primarily Fe^2+^) in H9C2 cells. The cells were seeded onto 6‐cm cell culture dishes at a density of 2 × 10^5^/each well. At the end of the processing, the cells were prepared by removing the supernatant and washing three times with PBS. Subsequently, a staining solution containing 1 μM of FerroOrange was added to the cells, and they were incubated for 30 min in an incubator. Subsequently, the fluorescent signals (*E*_*m*_ = 532 nm) were observed under a fluorescence microscope.

### 2.20 ROS levels of H9C2 cells

Briefly, H9C2 cells were seeded at a density of 1 × 10^4^ cells per well into 96‐well culture plates 1 day before treatment. After removing the cell culture solution and adding appropriate volume of DCFH‐DA (10 μM), the cells were incubated in a humidified incubator for 20 min. Subsequently, the cells were washed three times with PBS, and the fluorescence intensity was quantified using a multifunctional microplate reader (*E*_*x*_/*E*_*m*_ = 488/525 nm).

### 2.21 Mitochondrial complex activity assay

The treated H9C2 cells were collected, and mitochondria were obtained following the manufacturer's instructions. The absorbance of mitochondrial complexes I and II was measured at 340 and 605 nm, respectively. Viability values were calculated according to the manufacturer's instructions.

### 2.22 Mitochondrial membrane potential and apoptosis detection

The cells were seeded onto 6‐cm cell culture dishes at a density of 2 × 10^5^/per well. Before staining, the cells were prepared by removing the supernatant and washing thrice with PBS. Annexin V‐FITC and MitoTracker Red CMXRos staining solutions were added sequentially according to the manufacturer's instructions and incubated for 30 min at RT in the dark. Finally, fluorescent signals were observed under a fluorescence microscope, with red fluorescence for MitoTracker Red CMXRos and green fluorescence for Annexin V‐FITC.

### 2.23 Annexin V‐FITC/PI double staining for apoptosis

After treatment of the cells in each group, adherent cells were digested with EDTA‐free trypsin, washed twice with pre‐cooled PBS and centrifuged to collect the precipitates. The cells were resuspended with 500 μL of binding buffer, and then 5 μL of Annexin V and PI were added, respectively, and the cells were incubated for 15 min in the dark. Apoptosis was detected using flow cytometry (FC 500 MCL, Beckman Coulter, CA, USA).

### 2.24 Statistical analysis

All experimental data were analysed using SPSS 23.0. The experimental data were expressed as mean ± SEM, and statistically analysed using one‐way ANOVA followed by the LSD post hoc *test*. The significance level was set at *p* ≤ 0.05. Statistical maps were drawn using Prism 8.0 software (GraphPad Software, La Jolla, CA, USA).

## 3 RESULTS

### 3.1 Construction of FPN1‐overexpressing mice

**FIGURE 1:**
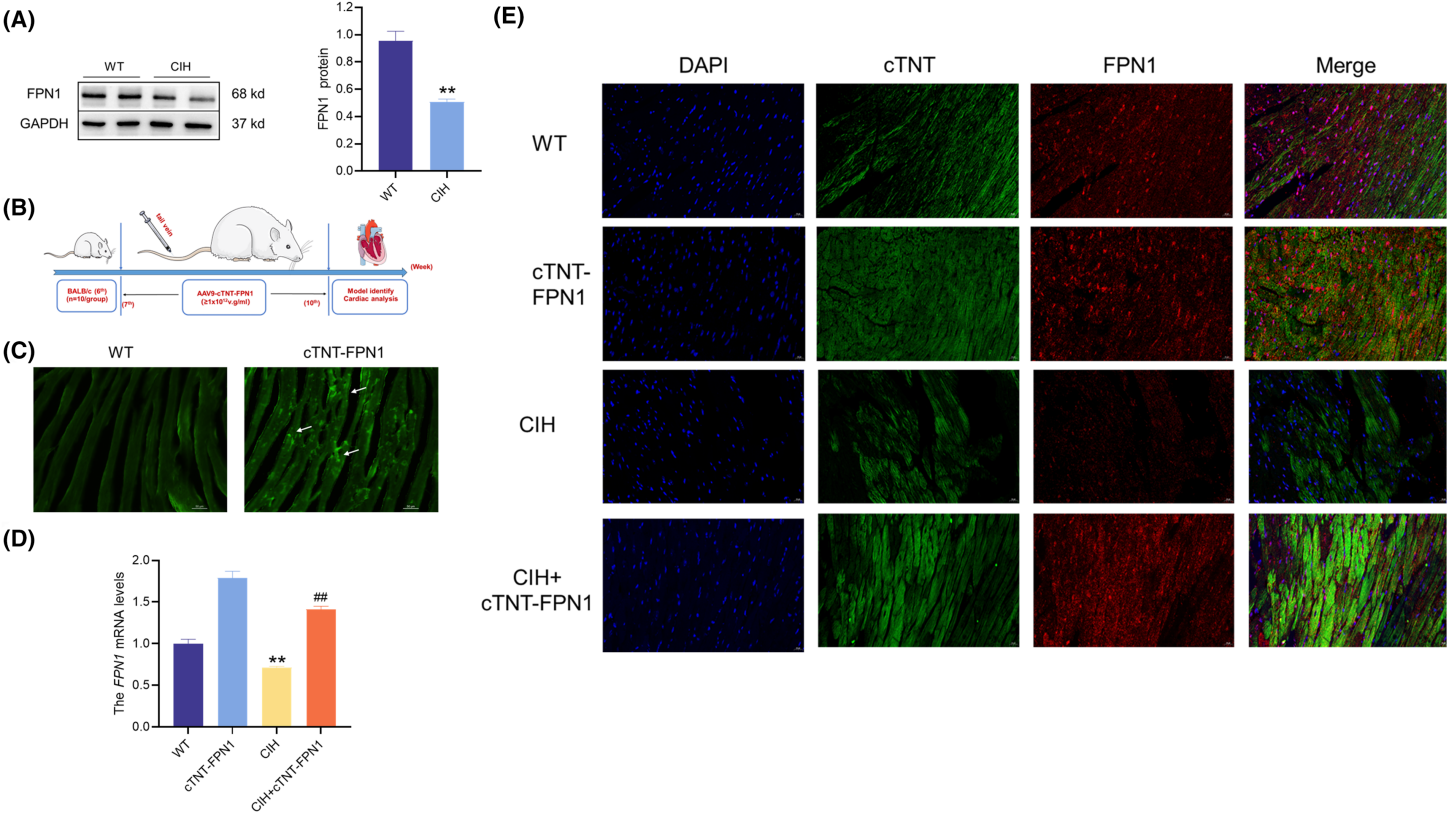
Construction of FPN1 overexpression mice. (A) The expression and statistics of FPN1 protein levels after CIH (*n* = 6). (B) The schematic diagram for the preparation of FPN1 overexpressing mice. (C) The fluorescent images of mice at the end of 10th week (scale bar = 50 μm, *n* = 3). (D) The expression and statistics of *FPN1* mRNA levels in heart tissue (*n* = 6). (E) The fluorescence intensity of FPN1 and cTNT in cardiomyocytes after CIH exposure (scale bar = 20 μm, *n* = 3). The data are presented as the means ± SEM. **p < 0.01 versus WT group. ^*##*^ *p* < 0.01 versus CIH group.

The expression of FPN1 protein in the mouse heart tissues decreased in the CIH model (Figure 1A). To further investigate the role of FPN1 in CIH‐induced myocardial injury, we constructed an FPN1 overexpressing mouse (Figure 1B). Overexpressing mice were successfully constructed by detecting the fluorescence intensity at end of Week10 (Figure 1C). Simultaneously, Q‐PCR results demonstrated a significant increase in *FPN1* mRNA levels in the cTNT‐FPN1 mice (Figure 1D). Double immunofluorescence labelling demonstrated that FPN1 was specifically expressed in cardiomyocytes of the cTNT‐FPN1 group (Figure 1E). These results indicated that mice were successfully constructed to overexpress FPN1 in cardiomyocytes.

### 3.2 Overexpression of FPN1 attenuated cardiac dysfunction and pathological damage in CIH mice

**FIGURE 2:**
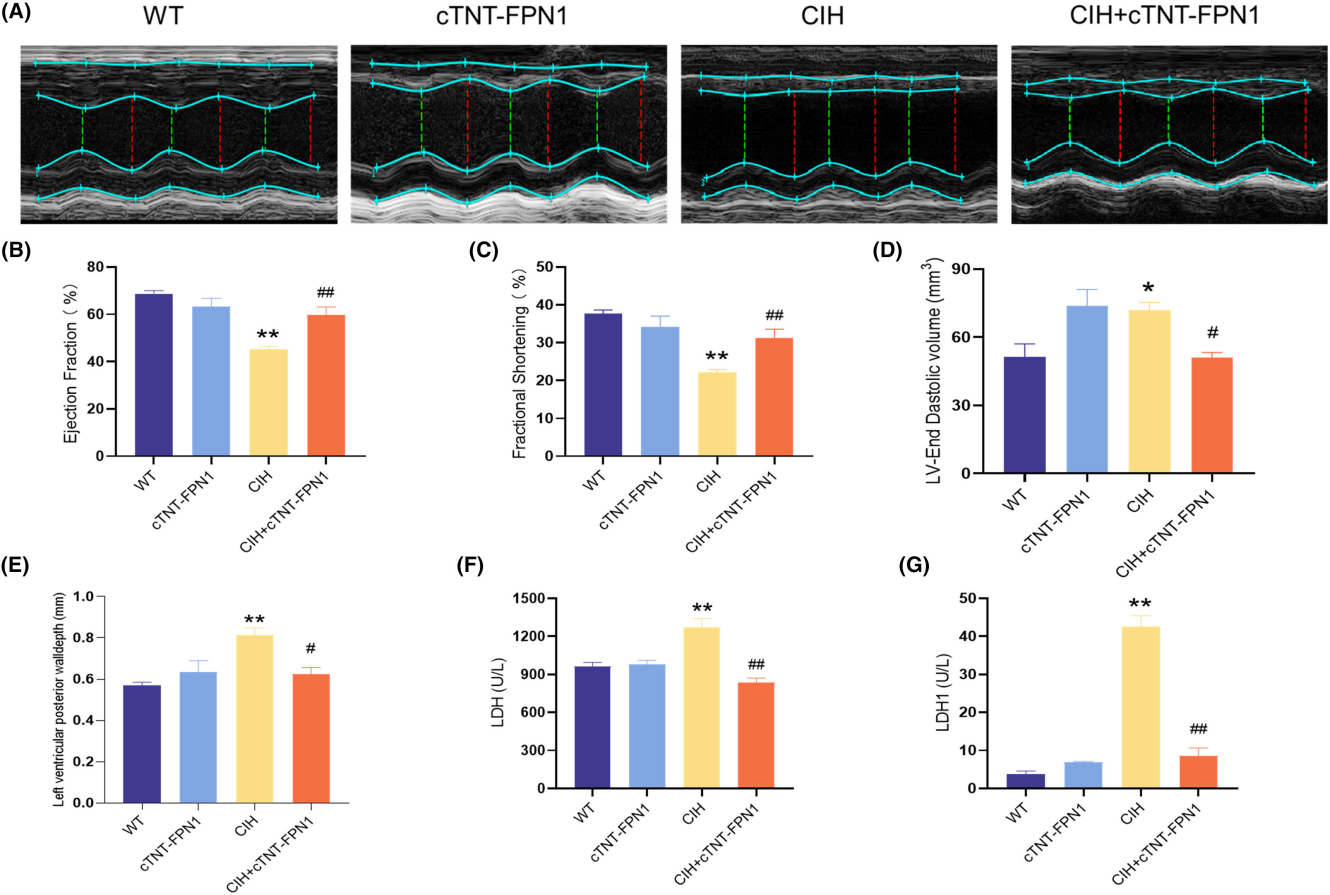
Overexpression of FPN1 attenuated cardiac dysfunction in CIH mice. (A) M‐model echocardiography in mice. (B) The ejection fraction of the left ventricle. (C) The fractional shortening. (D) The left ventricular end systolic volume. (E) The left ventricular posterior wall depth. (F, G) The serum LDH and LDH1 content. The data are presented as the means ± SEM. *n* = 6. *p < 0.05, **p < 0.01 versus WT group. ^*#*^ *p* < 0.05, ^*##*^ *p* < 0.01 versus CIH group.

The systolic and diastolic functions were assessed using a representative M‐mode parasternal short‐axis view (Figure 2A). After exposure to CIH, EF and FS decreased, whereas LVEDV increased (Figure 2B–D). In addition, the CIH group showed a significant increase in the LVPW, a characteristic index of myocardial hypertrophy (Figure 2E). Conversely, the CIH + cTNT‐FPN1 group showed significant improvements in these indicators (Figure 2B–E). The serum levels of LDH and LDH1 were significantly elevated in the CIH group but significantly decreased in the cTNT‐FPN1+ CIH group (Figure 2F,G).

**FIGURE 3:**
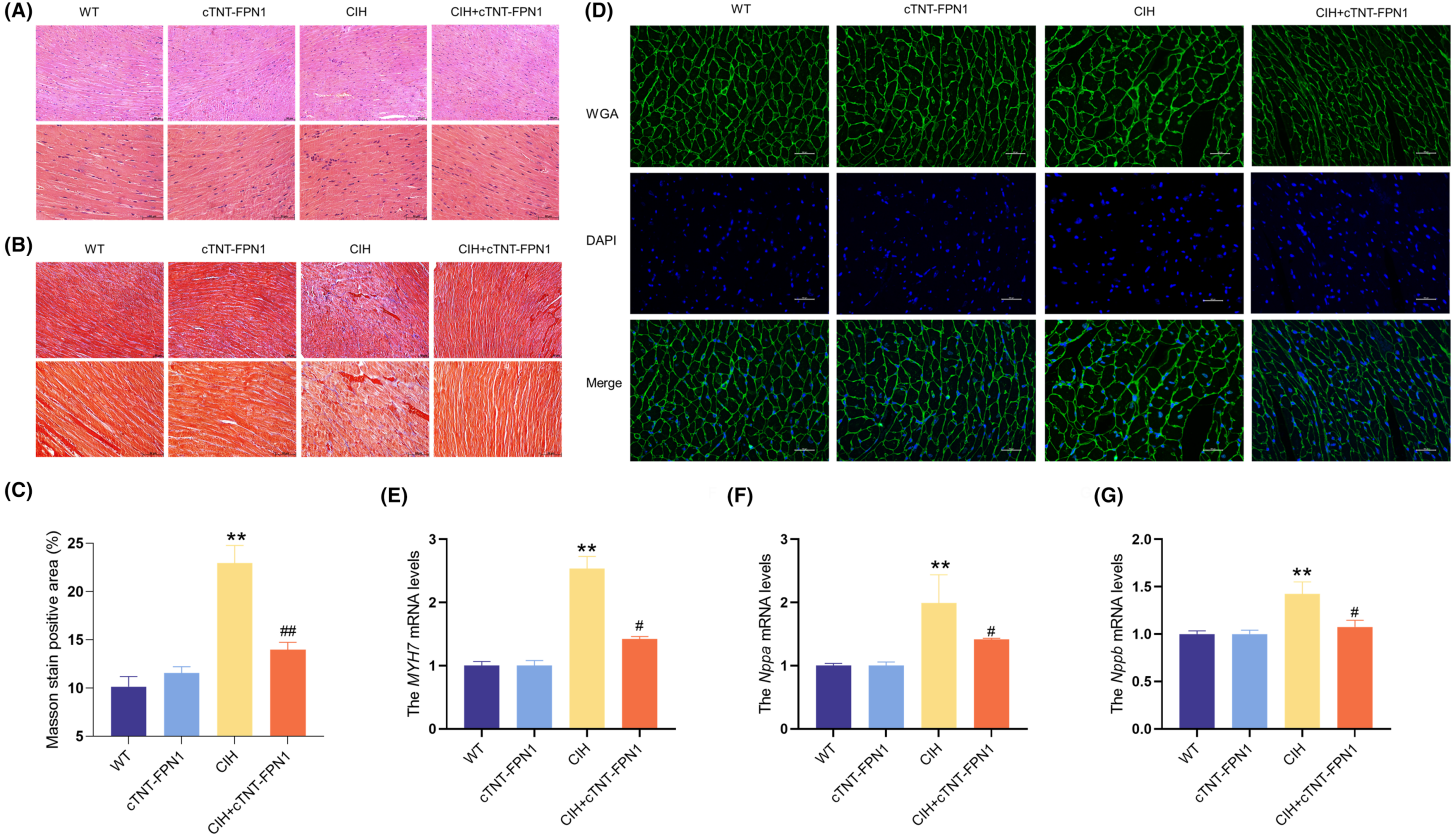
Overexpression of FPN1 attenuated cardiac hypertrophy and pathological damage in CIH mice. (A) The HE staining in heart tissue of different groups (scale bar = 50 μm, *n* = 3). (B) The Masson staining in heart tissue of different groups (scale bar = 50 μm, *n* = 3). (C) The WGA staining in heart tissue of different groups (scale bar = 100 μm, *n* = 3). (D–G) The *MYH7*, *Nppa* and *Nppb* mRNA levels in heart tissue (*n* = 6). The data are presented as the means ± SEM. **p < 0.01 versus. WT group. ^*#*^ *p* < 0.05, versus CIH group.

Histological analysis using HE and Masson staining revealed swollen and disorganized cardiomyocytes with inflammatory infiltrates and myocardial fibrosis in CIH mice compared to WT mice (Figure 3A–C). Conversely, mice in the cTNT‐FPN1 + CIH group showed improved myocardial structure and reduced fibrosis (Figure 3A–C). WGA staining showed that the cross‐sectional area of the cardiomyocytes in the CIH group was significantly larger than that in the WT group (Figure 3D). However, this effect was attenuated in the CIH + cTNT‐FPN1 group, as shown in Figure 3D. Furthermore, Q‐PCR results showed that the levels of *MYH7*, *Nppa* and *Nppb* mRNA, which are indicators of cardiac hypertrophy, were significantly increased in the CIH group and subsequently reduced in the CIH + cTNT‐FPN1 group (Figure 3E–G). Collectively, these findings suggest that exposure to CIH leads to myocardial hypertrophy, whereas FPN1 overexpression attenuates the associated pathological effects.

### 3.3 Overexpression of FPN1 alleviated CIH‐induced mitochondrial dysfunction and apoptosis

**FIGURE 4:**
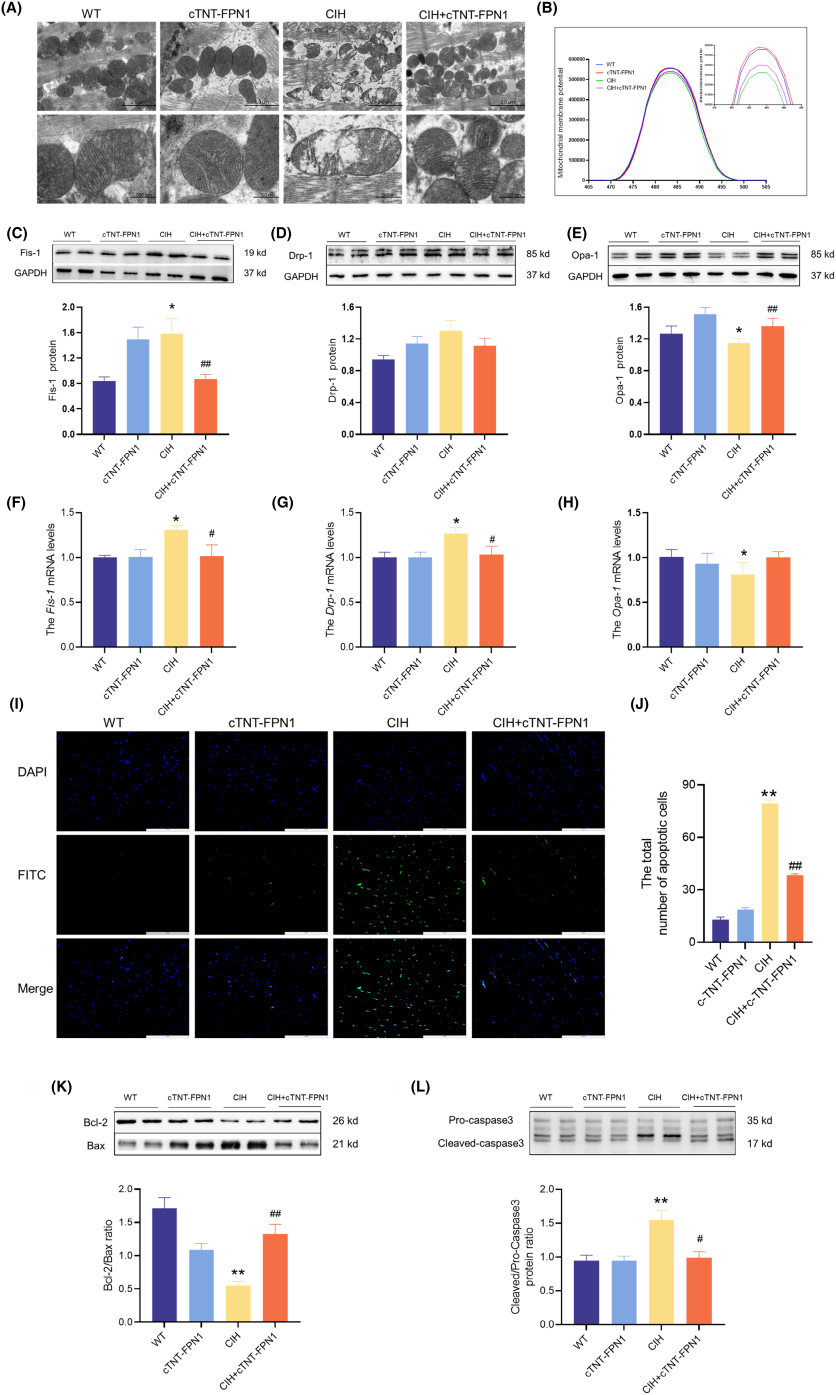
Overexpression of FPN1 alleviated the mitochondrial dysfunction and apoptosis induced by CIH. (A) The TEM images of mitochondria in the heart (scale bar = 2 μm or 500 nm, *n* = 3). (B) The mitochondrial membrane potential (*n* = 6). (C–E) The expression and statistics of Fis‐1, Drp‐1 and Opa‐1 protein levels in heart tissue (*n* = 6). (F–H) The *Fis‐1*, *Drp‐1* and *Opa‐1* mRNA levels in heart tissue (*n* = 6). (I) The TUNEL staining (scale bar = 100 μm, *n* = 3). (J) The total number of apoptotic cells is shown in panel I (*n* = 3). (K, L) The ratios and statistics of Bcl‐2/Bax and cleaved‐caspase 3/pro‐caspase 3 ratios in heart tissue detected by western blot (*n* = 6–8). The data are presented as the means ± SEM. *p < 0.05, **p < 0.01 versus WT group. ^*#*^ *p* < 0.05, ^*##*^ *p* < 0.01 versus CIH group.

TEM images revealed noticeable impairment and structural incompleteness of the mitochondrial spines in the myocardium of CIH mice compared with normal mice (Figure 4A). Conversely, mice in the CIH + cTNT‐FPN1 group showed a relatively intact mitochondrial structure in the heart compared to the CIH mice (Figure 4A). The JC‐1 probe was used to assess the MMP. As shown in Figure 4B, MMP levels were significantly decreased in the CIH group, which was improved in the cTNT‐FPN1 group. Western blot analysis revealed increased Fis‐1 and Drp‐1 protein levels and decreased Opa‐1 protein levels in the CIH group (Figure 4C–E). Conversely, in the cTNT‐FPN1 group, the increased Fis‐1 levels and decreased Opa‐1 levels returned to near‐normal levels (Figure 4C–E). The mRNA levels of *Fis‐1*, *Drp‐1* and *Opa‐1* showed a trend similar to that of their protein levels (Figure 4F–H).

Apoptosis was assessed in the myocardial tissue. The images showed that the CIH group exhibited a notable increase in the number of apoptosis‐positive cells (Figure 4I,J), as well as in the decresed ratios of Bcl‐2/Bax and incresed ratios of cleaved‐caspase 3/pro‐caspase 3 (Figure 4K,L) compared to the Con group. However, FPN1 overexpression in CIH mice resulted in the decreased apoptosis (Figure 4I–L). These results indicated that FPN1 overexpression effectively attenuated CIH‐induced mitochondrial damage and apoptosis in cardiac tissue.

### 3.4 Overexpression of FPN1 efficiently inhibited CIH‐induced oxidative stress in cardiac tissue

**FIGURE 5:**
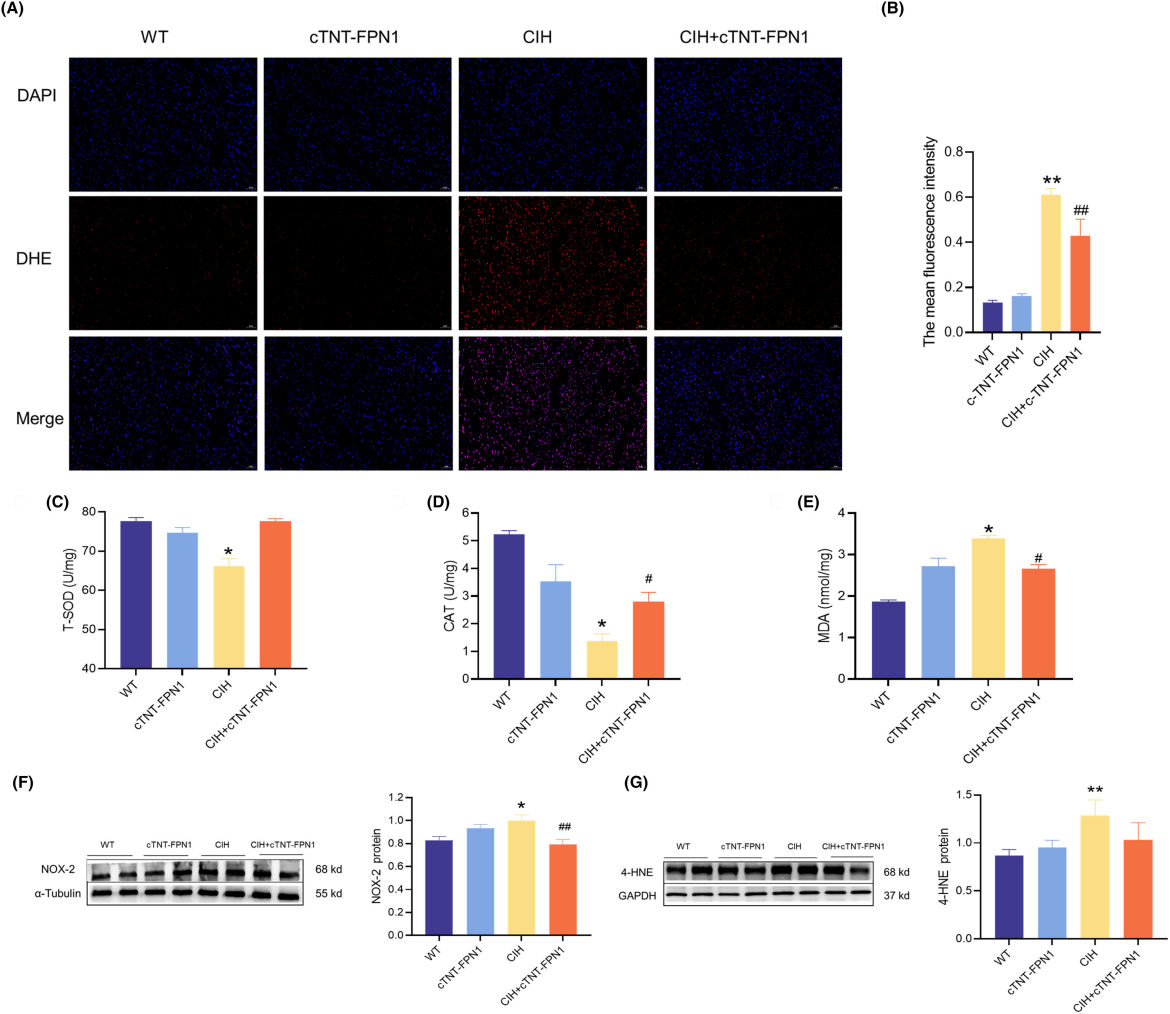
Overexpression of FPN1 efficiently inhibited oxidative stress in cardiac tissue induced by CIH. (A) The DHE staining in heart tissue (scale bar = 50 μm, *n* = 3). (B) The mean fluorescence intensity of DHE is shown in panel A (*n* = 3). (C–E) The activities of T‐SOD and CAT, and MDA content in cardiac tissues (*n* = 6). (F, G) The expression and statistics of NOX‐2 and 4‐HNE protein levels (*n* = 6). The results are presented as the mean ± SEM. *p < 0.05, **p < 0.01 versus WT group. ^*#*^ *p* < 0.05, ^*##*^ *p* < 0.01 versus CIH group.

Mitochondrial dysfunction is closely associated with an imbalance in oxidative stress. Therefore, we assessed oxidative stress levels in heart tissues. As shown by DHE staining, CIH mice exhibited a significant increase in ROS levels, which were reduced in the cTNT‐FPN1 group (Figure 5A,B). Simultaneously, T‐SOD and CAT activities decreased and MDA levels increased after CIH exposure, which were restored to normal levels in the CIH+cTNT‐FPN1 group (Figure 5C–E). Western blot analysis indicated that the expression of NOX‐2 and 4‐HNE proteins was significantly increased in the heart tissue of the CIH group compared to that in the Con group (Figure 5F,G). However, in the CIH + cTNT‐FPN1 group, these protein levels were reversed. Collectively, these results suggest that FPN1 overexpression mitigates oxidative stress following exposure to CIH.

### 3.5 Overexpression of FPN1 decreased iron deposits in the cardiac tissue of CIH mice

**FIGURE 6:**
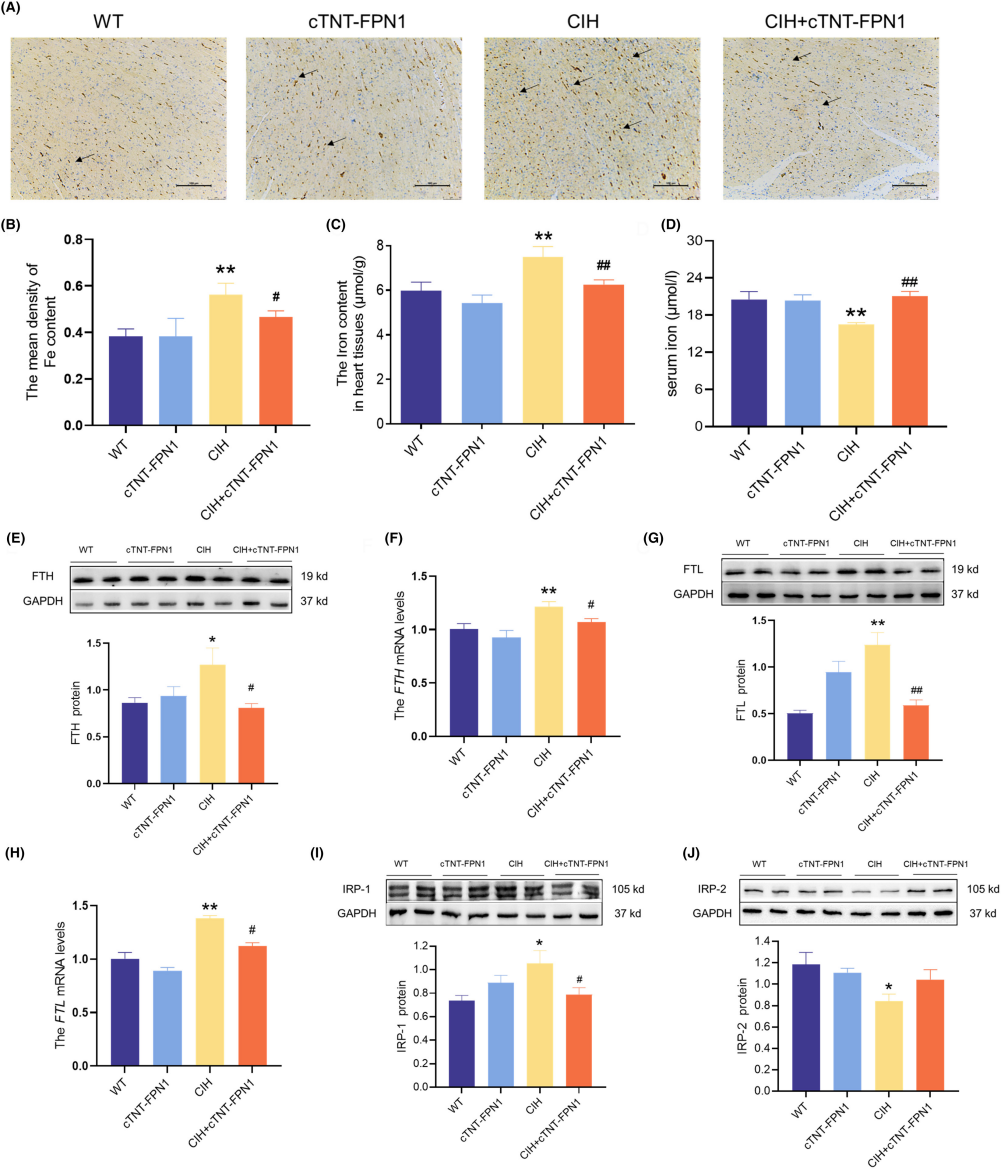
Overexpression of FPN1 weakened iron deposits in the cardiac tissue of CIH mice. (A) The Perls' staining of heart tissue (scale bar = 100 μm, *n* = 3). (B) The mean density of Fe content is shown in panel A (*n* = 3). (C) The total iron content in the cardiac tissue (*n* = 6). (D) The serum iron content (*n* = 6). (E, F) The expression and statistics of FTH protein and mRNA levels (*n* = 6). (G, H) The expression and statistics of FTL protein and mRNA levels in heart tissue (*n* = 6). (I, J) The expression and statistics of IRP‐1 and IRP‐2 protein levels (*n* = 6). The results are presented as the mean ± SEM. *p < 0.05, **p < 0.01 versus WT group. ^*#*^ *p* < 0.05, ^*##*^ *p* < 0.01 versus CIH group.

Excessive iron levels stimulate ROS production and exacerbate oxidative damage. The levels of trivalent and total iron in the cardiac tissue increased after exposure to CIH, as shown in Figure 6A–C. Simultaneously, serum iron levels decreased in the CIH group (Figure 6D), indicating abnormal systemic iron metabolism. Furthermore, the CIH group exhibited expression of iron storage proteins and genes, particularly FTH and FTL (Figure 6E–H). Western blot analysis revealed that the protein levels of IRP‐1 and IRP‐2 increased and decreased, respectively, in the CIH group (Figure 6I,J). Taken together, these results indicate that exposure to CIH induces iron accumulation in cardiac tissues. However, FPN1 overexpression attenuates iron deposition.

### 3.6 Hepcidin‐FPN1 involved in the iron deposition during CIH

**FIGURE 7:**
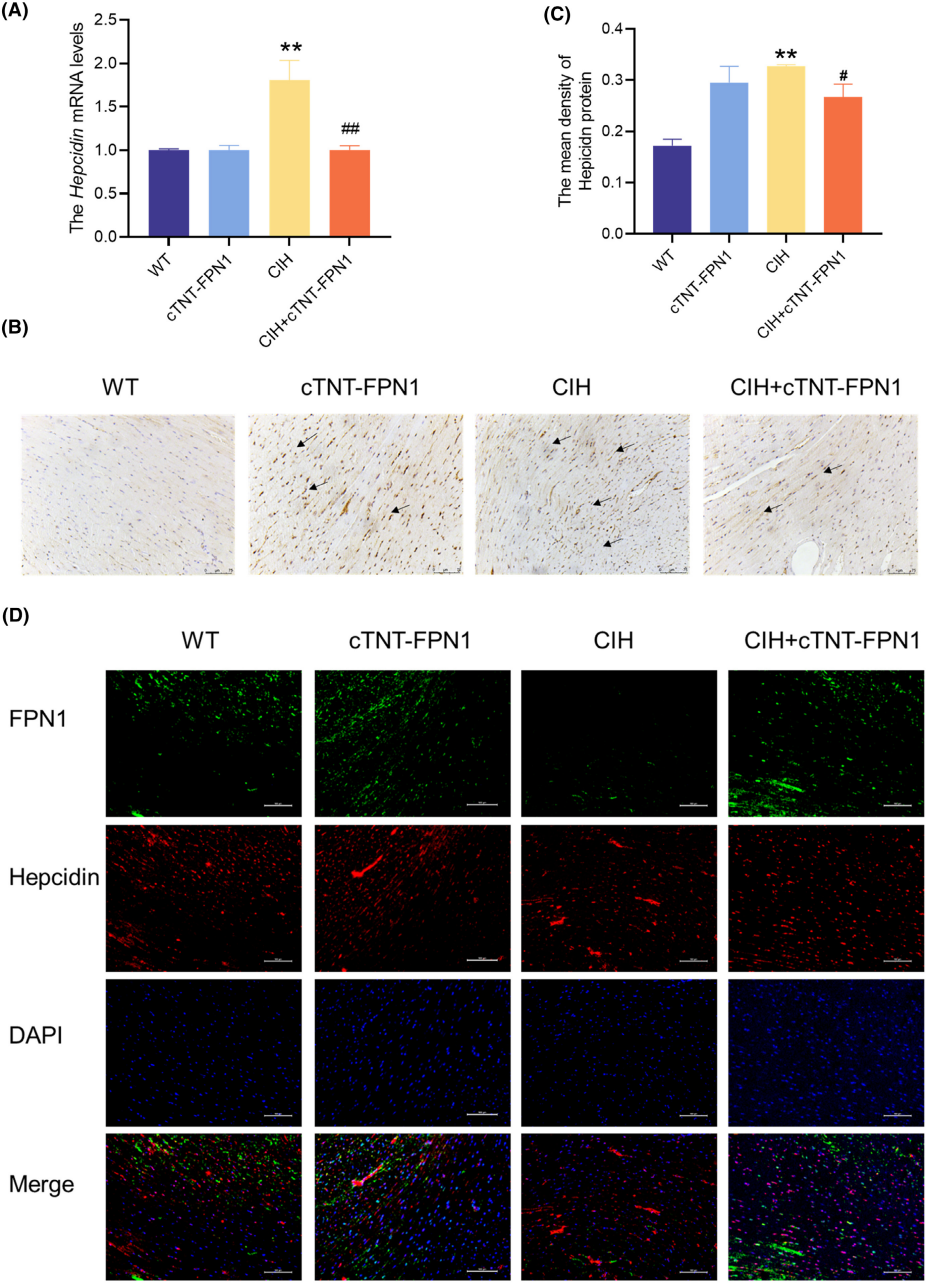
Hepcidin‐FPN1 involved in the iron deposition during CIH. (A) The *hepcidin* mRNA levels in heart tissue (*n* = 6). (B) The immunohistochemical staining of hepcidin protein (scale bar = 75 μm, *n* = 3). (C) The mean density of hepcidin content is shown in panel B (*n* = 3). (D) The immunofluorescence double label staining of FPN1 and hepcidin in heart tissue (scale bar = 25 μm, *n* = 3). The results are presented as the mean ± SEM. **p < 0.01 versus WT group. ^*#*^ *p* < 0.05, ^*##*^ *p* < 0.01 versus CIH group.

Subsequently, we explored the potential mechanisms underlying the interactions between hepcidin and FPN1 during CIH. We assessed hepcidin expression at both the transcriptional and translational levels. Q‐PCR analysis revealed an increase in *hepcidin* mRNA levels and IHC images showed an upregulation of hepcidin protein levels during CIH (Figure 7A–C). In addition, we used a fluorescent double labelling method to examine the co‐expression of hepcidin and FPN1 and observed that the increase in hepcidin was accompanied by a decrease in FPN1 expression in the CIH group (Figure 7D). The FPN1 overexpressing mice treated with CIH showed a decrease in elevated hepcidin levels compared to the CIH group.

### 3.7 The iron deposition in IH‐exposed H9C2 cells

**FIGURE 8:**
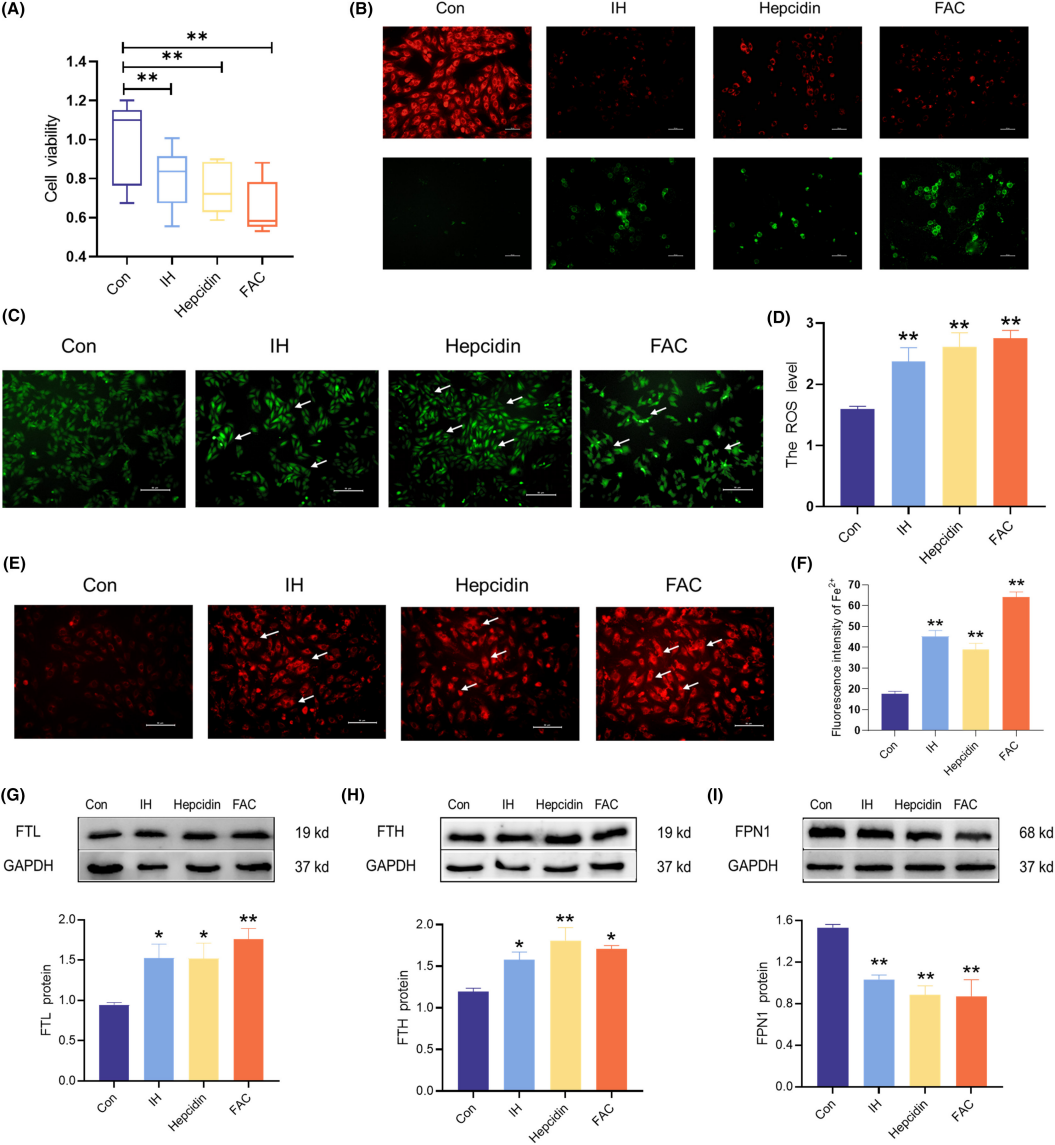
The iron deposition and ROS levels in H9C2 cells when exposed to IH. (A) The cell viability of H9C2 cells treated with IH, hepcidin, and FAC (*n* = 6). (B) The mitochondrial membrane potential and apoptosis in H9C2 cells (scale bar = 100 μm, *n* = 3). Red fluorescent labelling represents living cells that maintain mitochondrial membrane potential, green fluorescent labelling represents apoptotic living cells. (C, D) ROS level detection and analysis by DCFH‐DA probe in H9C2 cells (scale bar = 50 μm, *n* = 3). (E, F) The Fe^2+^ level detection and analysis by FerroOrange probe in H9C2 cells (scale bar = 50 μm, *n* = 3). (G–I) The expression and statistics of FTL, FTH, and FPN1 protein levels (*n* = 3). The results are presented as the mean ± SEM. *p < 0.05, **p < 0.01 versus con group.

To further investigate changes in iron metabolism, an IH model was established in vitro using H9C2 cells. Concurrently, H9C2 cells were treated with FAC and hepcidin to simulate high iron levels. The viability of H9C2 cells decreased after exposure to IH, hepcidin, or FAC treatment (Figure 8A). MMP and apoptosis probe analyses showed a decrease in MMP levels (red) and an increase in apoptosis (green) after treatment with IH, hepcidin, or FAC (Figure 8B). DCFH‐DA probe staining revealed an increase in the ROS levels induced by IH, hepcidin, and FAC (Figure 8C,D). Additionally, FerroOrange probe staining showed a significant increase in LIP after treatment in the IH, hepcidin, and FAC groups (Figure 8E,F). Western blot analysis revealed that the IH, hepcidin, and FAC groups exhibited increased levels of FTL and FTH, whereas FPN1 levels were decreased (Figure 8G–I). These results indicate that IH effectively promoted intracellular iron levels and ROS, similar to the effects of FAC. Notably, administration of exogenous hepcidin further exacerbated cellular damage in the presence of iron overload. These observations are consistent with our in vivo results, suggesting that elevated hepcidin and reduced FPN1 levels accelerate the progression of myocardial cell damage during IH.

### 3.8 Elevated hepcidin contributed to FPN1 decrease and iron deposition in IH‐exposed H9C2 cells

**FIGURE 9:**
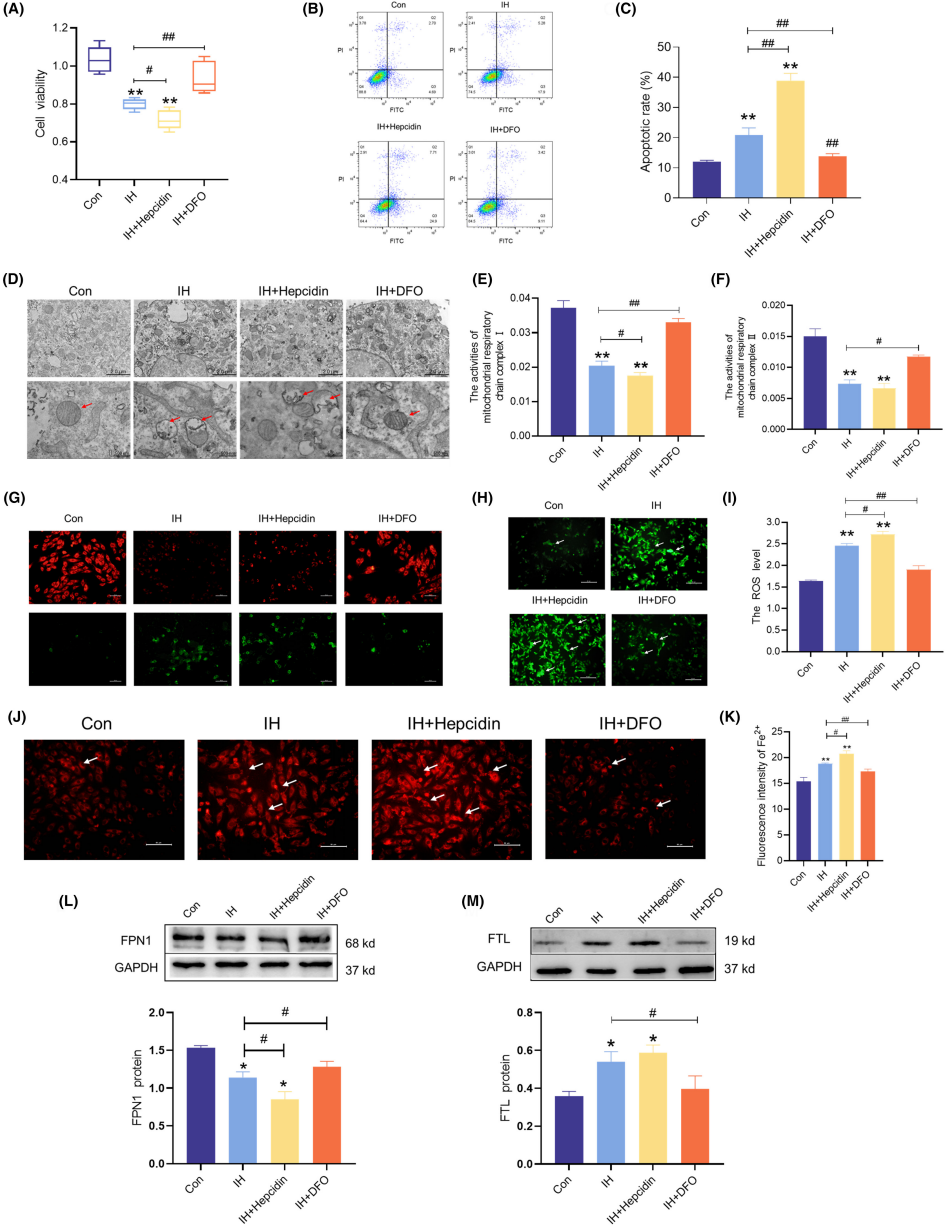
The elevated hepcidin contributed to FPN1 decrease and iron deposition in H9C2 cells exposed to IH. (A) The cell viability was treated with IH, IH + hepcidin and IH + DFO (*n* = 5). (B) Detection of apoptosis by flow cytometry (*n* = 3). (C) The statistics of apoptosis as shown in panel B (*n* = 3). (D) The TEM images of mitochondria in H9C2 cells (*n* = 3). (E, F) The activities of mitochondrial respiratory chain complex I and II (*n* = 6 or 3). (G) The mitochondrial membrane potential and apoptosis in H9C2 cells (scale bar = 100 μm, *n* = 3). Red fluorescent labelling represents living cells that maintain mitochondrial membrane potential, green fluorescent labelling represents apoptotic living cells. (H, I) ROS level detection and analysis by DCFH‐DA probe in H9C2 cells (scale bar = 50 μm, *n* = 3). (J, K) The Fe^2+^ level detection and analysis by FerroOrange probe in H9C2 cells (scale bar = 50 μm, *n* = 3). (L, M) The expression and statistics of FPN1 and FTL protein levels (*n* = 4). The results are presented as the mean ± SEM. *p < 0.05, **p < 0.01 versus con group. ^*#*^ *p* < 0.05, ^*##*^ *p* < 0.01 versus IH group.

To evaluate the role of hepcidin in IH, we administered exogenous hepcidin during IH treatment. CCK8 assay results showed a significant reduction in cell viability in the IH + hepcidin group, which was further confirmed by flow cytometry (Figure 9A–C). However, DFO treatment improved viability and reduced apoptosis in IH‐treated H9C2 cells (Figure 9A–C). TEM images revealed clear impairment and structural incompleteness in the mitochondrial spines of the IH and IH + hepcidin groups compared to those in the Con group (Figure 9D). The activities of mitochondrial respiratory chain complexes I and II were decreased in the IH model and hepcidin‐treated groups (Figure 9E,F). Furthermore, consistent with the results shown in Figure 8, the DCFH‐DA probe, FerroOrange probe, and MMP levels showed similar trends in the IH model, with the damage further exacerbated in the IH + hepcidin model (Figure 9G–K). Western blotting results showed that the exogenous administration of hepcidin aggravated the IH‐induced decrease in FPN1 and increase in FTL (Figure 9L,M). However, the cellular damage and elevated iron levels were reversed with DFO administration. These results indicate that exogenous administration of hepcidin exacerbates IH‐induced damage in H9C2 cells; however, this damage could be mitigated using an iron chelator.

## 4 DISCUSSION

LVH has received increasing attention as a cardiovascular complication resulting from repetitive airway obstruction events. OSA contributes to an increase in left ventricular mass (LVM) and the development of LVH. Patients with LVH have an increased incidence of cardiovascular disease and mortality.^3^, ^30^ In addition, our research has demonstrated that exposure to CIH leads to an increase in ventricular wall thickness and significant changes in the myocardial structure in mice.^31^ Patients with OSA experience recurrent episodes of CIH, leading to an increase in left ventricular transmural pressure and cardiac afterload, ultimately resulting in cardiac hypertrophy.^32^, ^33^ Physiological cardiac hypertrophy has the potential to restore ventricular wall tone to normal levels. However, if the underlying cause is persistent and excessive, it may progress to pathological cardiac hypertrophy and ultimately lead to heart failure.^34^ Therefore, it is imperative to take proactive measures to prevent and treat early stages of chronic excessive hypertrophy. The present study demonstrated that the exposure of mice to CIH for 4 weeks led to the development of cardiac hypertrophy and dysfunction. This was evidenced by the observed increase in the cross‐sectional area by WGA staining and the significant increase in the *MYH7* gene, which was specifically associated with hypertrophic cardiomyopathy.^35^

Mitochondrias play crucial roles as organelles and energy sources in cardiomyocytes and exert a significant impact on the progression of cardiac hypertrophy. Impairments in mitochondrial structure and function disrupt energy metabolism, consequently inducing cardiac hypertrophy.^36^ The inhibition of mitochondrial division has also been proposed to attenuate cardiac injury induced by CIH.^37^ Mitochondrias maintain their morphology and distribution through ongoing processes of division and fusion. Dysregulation of these processes results in morphological and structural fragmentation and aggregation, triggering an apoptotic cascade.^38^ Apoptosis is thought to play a significant role in inducing CIH‐related cardiac hypertrophy because cardiomyocytes are unable to divide and apoptosis of these cells places an increased workload on the remaining cells.^39^ The present study confirms previous findings that CIH‐induced cardiac hypertrophy exhibits notable upregulation of pro‐apoptotic cytokines and their receptors.^40^

ROS play a significant role in inducing phenotypic adverse effects in experimental animal models of CIH and patients with OSA.^41^ The development of cardiac hypertrophy is strongly associated with mitochondrial oxidative stress and extensive research has demonstrated that inhibiting ROS production can effectively ameliorate myocardial hypertrophy.^42^, ^43^ Additionally, inhibition of mitochondrial protein synthesis during myocardial ischaemia/reperfusion (I/R) may mitigate oxidative stress‐induced cardiac injury.^44^ Furthermore, existing evidence indicates that ROS can directly trigger apoptosis and facilitate the progression of cardiac hypertrophy.^45^

Iron is an essential transition metal that plays a vital role in maintaining optimal mitochondrial function by participating in energy metabolism.^46^, ^47^ Cardiomyocytes with high activity levels rely on iron for their metabolic processes.^48^ High oxygen turnover in the heart requires a delicate balance between adequate iron supply and strict regulation of LIP to prevent excessive ROS production.^49^ Clinical trials have demonstrated that patients diagnosed with heart failure frequently exhibit iron deficiency and that even mild iron deficiency can impair cardiac function.^50^, ^51^ Conversely, excess iron can impair cardiac function, as evidenced by heart failure due to iron overload in patients with β‐thalassaemia and haemochromatosis.^52^, ^53^ Cardiomyocytes acquire iron predominantly through the Tf‐TfR1 system, with excess iron entering the cytoplasm and being stored in ferritin within the mitochondrial compartment.^54^ FPN1 is the only recognized mammalian iron export protein that can release iron into the circulatory system via iron recycling, absorption and storage sites.^23^ However, it is important to note that downregulation of TfR1 alone does not effectively prevent iron overload in hearts lacking FPN1, suggesting that FPN1‐mediated iron release plays a critical role in maintaining iron homeostasis in cardiomyocytes.^55^

FPN1 consists of two 6‐stranded transmembrane helical bundles, N and C lobes, connected by a cytoplasmic loop. The C and N ends are located in the cytoplasm, whereas the two helical bundles enclose a cavity that facilitates the release of iron from the cell.^18^ The regulation of FPN1 is influenced by transcription levels. The 5′ untranslated region (UTR) of *FPN1* mRNA contains an iron‐responsive element (IRE), which enables post‐transcriptional regulation of FPN1 expression through the interaction between IRPs and the IRE system.^21^, ^56^ Hepcidin, a liver‐derived antibacterial polypeptide, controls iron utilization by participating in the binding and internalization of FPN1.^52^ The importance of the hepcidin/FPN1 axis has been observed in the occurrence of systemic iron overload diseases such as haemochromatosis and β‐thalassaemia.^52^, ^53^

In cases of iron overload, the hepatic production and release of hepcidin is increased, leading to accelerated degradation of FPN1, which impedes the transport of iron into the bloodstream, resulting in a reduced supply of iron from the small intestinal epithelial cells and macrophages.^19^, ^57^ In addition, both FPN1 and hepcidin have been observed to be also expressed in cardiomyocytes.^58^ The hepcidin/FPN1 axis in the heart is critical for the maintenance of normal cardiac function and any disruption of this axis could potentially lead to severe cardiac metabolic and contractile dysfunction, even in the presence of intact systemic iron homeostasis.^59^, ^60^ During CIH, we observed changes in the levels of IRPs and upregulation of hepcidin expression in the cardiac tissue, as well as a decrease in FPN1 expression. Extremely high FPN1 expression theoretically leads to exocytosis of large amounts of intracellular iron into the bloodstream, causing iron deficiency in organisms or cells. However, we did not observe a significant decrease in the body iron levels after FPN1 overexpression, suggesting that the body responds to stimuli to prevent iron deficiency. A possible reason for this may be that reduced iron levels conversely stimulate low expression of hepcidin, resulting in its binding to FPN1 to ensure normal iron homeostasis.

Accumulating evidence supports the notion that the modulation of the hepcidin‐FPN1 axis is an important therapeutic strategy for regulating iron levels.^61^ Primary interventions include direct or indirect inhibition of hepcidin expression or prevention of FPN1 degradation using anti‐hepcidin antibodies, inhibitors of hepcidin production/synthesis or FPN1 agonists.^58^, ^59^ Nevertheless, many therapeutic strategies under development face significant obstacles that must be overcome before they can be used effectively. For example, the advancement of RNAi technologies requires improved design strategies to mitigate off‐target interactions and increase stability to achieve optimal half‐lives.^62^

While anti‐hepcidin antibodies showed good tolerability in Phase I clinical trials, these increases were only transient, returning to baseline after 8 days.^63^ In addition, although a high‐throughput screen found that the thiamine derivative furanethiamine inhibits feromodulin binding to FPN1, in vivo evaluation of furanethiamine did not affect FPN1 labile iron pool levels.^64^ AAV is a common single‐stranded DNA virus that can deliver specific gene loads without interfering with normal physiological functions and is widely used in gene therapy research and practical applications; AAV‐based gene therapy applications have led to several products entering clinical development.^65^ Therefore, we used an adenovirus to target FPN1 overexpression in vivo, which counteracted the deleterious effects of FPN1 overexpression induced by increased hepcidin expression. However, their use may raise a number of practical and theoretical safety issues; for example, toxicity studies on AAV delivery in animal experiments require further research.

In conclusion, our study demonstrates a potential association between increased iron levels in cardiomyocytes and the manifestation of oxidative stress and compromised mitochondrial function after exposure to CIH. Moreover, upregulation of FPN1 expression has been found to mitigate cellular iron accumulation, thereby alleviating CIH‐induced cardiac hypertrophy and mitochondrial impairment. These findings provide a rationale for the development of therapeutic interventions to address cardiovascular damage in individuals with OSA.

## AUTHOR CONTRIBUTIONS

**Ji‐Xian Song:** Conceptualization (equal); data curation (equal); writing – original draft (equal); writing – review and editing (equal). **Shan Xu:** Conceptualization (equal); data curation (equal); writing – original draft (equal). **Qi Chen:** Data curation (equal); methodology (equal). **Yujing Gou:** Data curation (equal); methodology (equal). **Chen‐Bing Zhao:** Data curation (equal); methodology (equal). **Cui‐Ling Jia:** Data curation (equal); methodology (equal). **Han Liu:** Data curation (equal); methodology (equal). **Zhi Zhang:** Data curation (equal); methodology (equal). **Bo‐liang Li:** Methodology (equal); software (equal). **Yuhui Gao:** Data curation (equal); methodology (equal). **Yashuo Zhao:** Conceptualization (equal); project administration (equal); visualization (equal); writing – original draft (equal); writing – review and editing (equal). **En‐Sheng Ji:** Conceptualization (equal); funding acquisition (equal); supervision (equal); writing – original draft (equal); writing – review and editing (equal).

## FUNDING INFORMATION

The Central Leading Local Science and Technology Development Fund Project (216Z7704G), and Hebei Administration of Traditional Chinese Medicine (Z2022005).

## CONFLICT OF INTEREST STATEMENT

The authors confirm that there are no conflicts of interest.

## Data Availability

The data utilized to substantiate the study's conclusions are provided in the article. The data underlying this article will be shared on reasonable request to the corresponding author.
